# Intelligent Bone Age Assessment: An Automated System to Detect a Bone Growth Problem Using Convolutional Neural Networks with Attention Mechanism

**DOI:** 10.3390/diagnostics11050765

**Published:** 2021-04-24

**Authors:** Mohd Asyraf Zulkifley, Nur Ayuni Mohamed, Siti Raihanah Abdani, Nor Azwan Mohamed Kamari, Asraf Mohamed Moubark, Ahmad Asrul Ibrahim

**Affiliations:** Department of Electrical, Electronic and Systems Engineering, Faculty of Engineering and Built Environment, Universiti Kebangsaan Malaysia, Selangor 43600, Malaysia; ayuni@siswa.ukm.edu.my (N.A.M.); raihanah.abdani@siswa.ukm.edu.my (S.R.A.); azwank@ukm.edu.my (N.A.M.K.); asrafmohamed@ukm.edu.my (A.M.M.); ahmadasrul@ukm.edu.my (A.A.I.)

**Keywords:** bone growth disorder, X-ray image, convolutional neural network, attention mechanism, regression network

## Abstract

Skeletal bone age assessment using X-ray images is a standard clinical procedure to detect any anomaly in bone growth among kids and babies. The assessed bone age indicates the actual level of growth, whereby a large discrepancy between the assessed and chronological age might point to a growth disorder. Hence, skeletal bone age assessment is used to screen the possibility of growth abnormalities, genetic problems, and endocrine disorders. Usually, the manual screening is assessed through X-ray images of the non-dominant hand using the Greulich–Pyle (GP) or Tanner–Whitehouse (TW) approach. The GP uses a standard hand atlas, which will be the reference point to predict the bone age of a patient, while the TW uses a scoring mechanism to assess the bone age using several regions of interest information. However, both approaches are heavily dependent on individual domain knowledge and expertise, which is prone to high bias in inter and intra-observer results. Hence, an automated bone age assessment system, which is referred to as Attention-Xception Network (AXNet) is proposed to automatically predict the bone age accurately. The proposed AXNet consists of two parts, which are image normalization and bone age regression modules. The image normalization module will transform each X-ray image into a standardized form so that the regressor network can be trained using better input images. This module will first extract the hand region from the background, which is then rotated to an upright position using the angle calculated from the four key-points of interest. Then, the masked and rotated hand image will be aligned such that it will be positioned in the middle of the image. Both of the masked and rotated images will be obtained through existing state-of-the-art deep learning methods. The last module will then predict the bone age through the Attention-Xception network that incorporates multiple layers of spatial-attention mechanism to emphasize the important features for more accurate bone age prediction. From the experimental results, the proposed AXNet achieves the lowest mean absolute error and mean squared error of 7.699 months and 108.869 months^2^, respectively. Therefore, the proposed AXNet has demonstrated its potential for practical clinical use with an error of less than one year to assist the experts or radiologists in evaluating the bone age objectively.

## 1. Introduction

**Figure 1:**
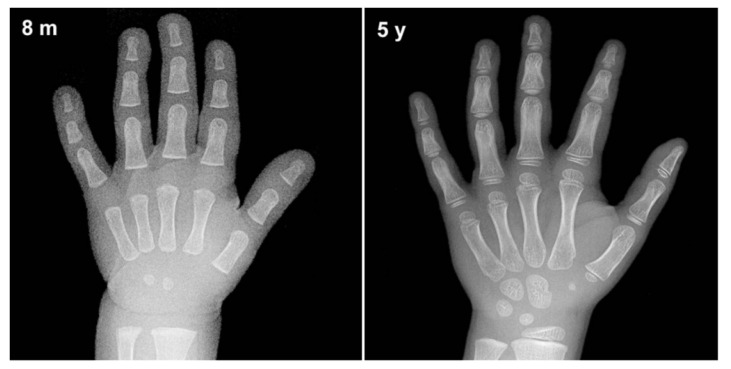
An example of bone changes between an 8-month-old baby and a 5-year-old toddler.

Skeletal bone age assessment is a standard procedure to assess the skeletal maturity or bone age in pediatric radiology. Bone age refers to the actual level of bone growth development, which can be assessed continuously as the skeleton bone grows, as such, it will change in shape and size over time [1]. Figure 1 illustrates an example of bone changes between an 8-month-old baby and a 5-year-old toddler. Therefore, there are significant changes as a toddler grows older until they reach the maturity age of 18 years, and thus a large discrepancy between the bone age and chronological age might reveal a growth problem [2]. Furthermore, a significant deviation from the normal growth pattern might indicate various health issues that include genetic disorders, hormonal problems, and endocrine disorders [3]. This assessment can also be used to facilitate the pediatricians in predicting a child’s adult height and puberty age. Additionally, it is also useful in monitoring growth hormone therapy progress for short patients and early diagnosis of pediatric endocrine disorders patients. Hence, bone age assessment is an imperative method in both pediatric endocrinology and orthopedics for assessing child skeletal system maturity [4].

The most prominent approach for bone age assessment is through X-ray images of the non-dominant hand and wrist by comparing them with a set of reference hand atlases of children of the same gender and age. The assessment through X-ray images can clearly provide a profound bone development pattern with minimum radiation exposure [5]. The assessment is done using a non-dominant hand due to the nature of bone ossification. It can be used to predict a child’s age until he reaches the end of the adolescence stage, where at this stage, the bone growth will stop completely. A recently published article by Guo et al. [6] has mentioned that an estimated 76% of radiologists have utilized hand X-ray images to assess the bone age. Moreover, X-ray images is a popular screening modality that has been widely used to screen various diseases like COVID-19 [7,8], pneumonia [9,10], osteoarthritis [11,12], dental decay [13,14], and many more. In addition, X-ray images have also been used as an imaging modality in astronomy to investigate the X-ray emission from celestial objects due to its advantages such as simplicity and high-speed [15,16,17].

Over the past years, the most popular approach to bone age assessment is done through the Greulich–Pyle (GP) or Tanner–Whitehouse (TW) methods. The GP approach [18] was introduced in 1959 to assess the bone age by subjectively comparing a whole X-ray image with a list of standard bone age atlas with varying bone maturity levels. Then, the corresponding bone age of a patient is chosen based on the closest matching template. The GP approach is easy and can be quickly implemented, whereby almost 80% of radiologists have utilized this method to assess the bone age according to an online survey performed by the Society of Pediatric Radiology [19]. However, the bone age assessment through this approach is challenging and suffered from a high bias in inter and intra-observer variability to accurately assess the bone age.

**Figure 2:**
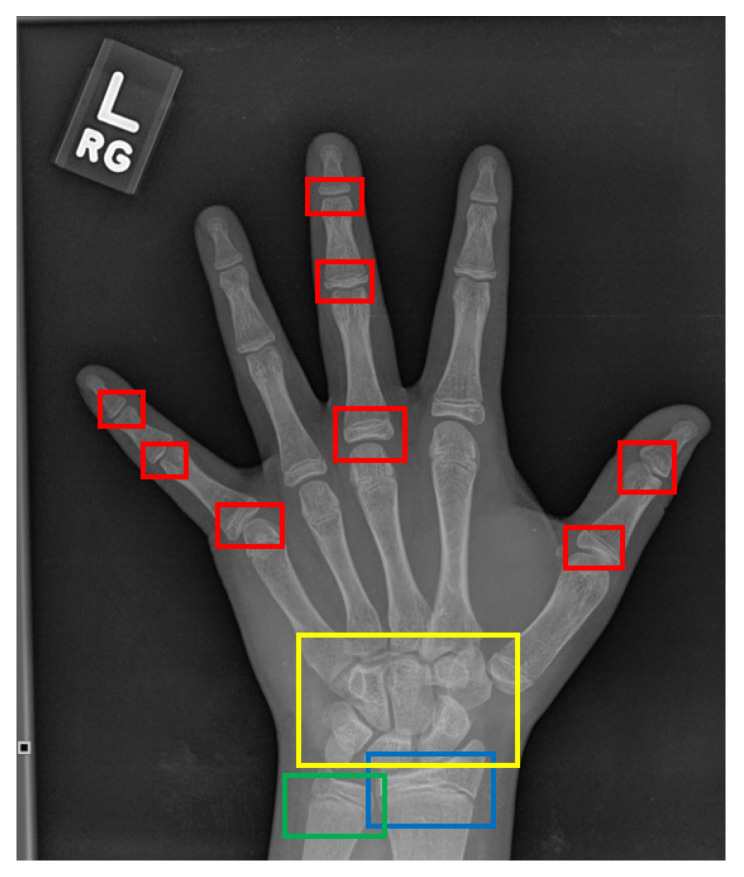
The ROIs of a hand X-ray image for the TW approach. The red boxes refer to the phalangeal joints, the yellow box refers to the carpal region, the blue box refers to the radius physis region, and the green box refers to the ulna physis region.

On the other hand, the TW approach [20] analyzes specific regions of interest (ROIs), which are assessed individually through a scoring mechanism, instead of assessing the bone age based on the whole X-ray image. The ROIs comprise of radius, ulna, carpal, and phalanges bones which are annotated as blue, green, yellow, and red boxes, respectively as depicted in Figure 2. The phalanges bones are only considered from three specific fingers which are thumb, index, and middle fingers. Then, each ROI is evaluated by a numerical score system and the final bone age is estimated by averaging all the ROIs’ scores. Moreover, the TW approach can predict bone age more accurately compared to the GP approach and it is also suitable for automation due to its modular structure. However, the strategy of the TW approach is comparatively complex which makes it seldomly used as compared to the GP approach [21]. Although both GP and TW approaches have their advantages, these methods are heavily dependent on the domain knowledge and expertise of the assessor. Furthermore, it is also noticeable that both approaches are time-consuming, where it takes an average of processing time of ≈7.9 and ≈1.4 min per image to complete the bone age assessment, respectively [21]. Nonetheless, the research in [1] has mentioned that an experienced radiologist requires around 30 min per image to assess the bone age through the TW approach.

## 2. Computerized Bone Age Assessment

Despite these drawbacks, more works based on computer-assisted systems have been developed to assist radiologists to predict the bone age faster. Generally, the earliest automated systems handle the bone age assessment as a regression problem that requires pre-processing steps such as hand segmentation, feature extraction, and regressor frameworks. Henceforth, high-quality X-ray images are indispensable for robust and accurate hand segmentation approaches. Moreover, the traditional regression frameworks such as support vector regression and neural networks require manual or hand-crafted features to predict the bone age which also leads to heavy dependence on the skills and experiences of the programmer.

In recent years, the proliferation and advent of deep learning through convolutional neural networks (CNNs) have captivated interest in various fields such as object detection [22], agriculture [23], and natural language processing [24] with prominent results. Particularly, the deep learning models have been adopted in many medical image analysis applications, namely physiotherapy [25], eye disease detection [26], and skin lesion segmentation [27]. This adoption is driven by the ability of deep learning in discovering multiple levels of discriminative features by automatically learn the high-level abstractions of the image data to avoid any feature engineering process. Hence, a complete end-to-end learning task is achieved and can efficiently solve the limitations of the conventional machine learning frameworks. Apart from that, the original hand X-ray images used in the automated bone age assessment systems were captured in various sizes and conditions, where the radiological markers are placed at different positions. Another noticeable limitation is the captured hand images are not properly aligned, where some images contain hand-posed that are tilted at certain angles. These limitations affect the screening time as it will take a longer time and require tedious pre-processing steps before the bone age can be predicted.

The early works that implement CNNs such as Spampinato et al. [28] and Iglovikov et al. [1] utilize a straightforward combination of multiple layers of convolution, normalization layer, and activation function. There is no residual or feed-forward layer applied, which makes the backpropagation update of the parameters prone to diminishing gradient problem. Similarly, the work by Lee et al. [29] has also explored several basic architectures that include AlexNet, GoogleNet, and VGG-16. Only GoogleNet utilizes a wider set of CNN filters with different combinations of kernel sizes. Hence, our previous [30] work has implemented a CNN network with residual skip connection, coupled with separable convolution through Xception architecture. However, the TW approach has proven that there are specific regions that are more informative in determining the bone age such as interphalangeal joints and carpal region. Therefore, an attention mechanism that allows the network to learn the strategic location on the images so that more weights will be allocated is integrated with the Xception architecture. The proposed model, which is termed as Attention-Xception Network (AXNet), encompasses two crucial modules which are the image normalization module and bone age prediction module.

The image normalization module is developed to properly align each hand X-ray image into a standardized form. The module will first segment the hand region so that only the masked hand region will later be processed. Then, the four key-points are determined to properly rotate the hand masked images in an upright position without being slanted either to the left or right side of the image. The masked and rotated images are then fed to the translational alignment unit so that the normalized image will be position in the middle of the image. The bone age regression module will then predict the bone age using the standardized X-ray images, which are fed into an Attention-Xception network that incorporates multiple attention mechanism layers. The main motivation of employing the attention mechanism is inlined with the TW approach, whereby several regions are more important in predicting the bone age compared to the others. Hence, the prediction accuracy of the network will be improved by allocating more weights to the attentioned regions. The proposed AXNet has been extensively trained, validated, and tested on a large dataset comprised of a total number of 12,811 images according to a ratio of 8:1:1, respectively to avoid the overfitting problem. Furthermore, the bone age prediction module also implements a three-layer residual separable convolution scheme to overcome the diminishing gradient problem during the training process. It is noteworthy to point out that the proposed AXNet does not exploit any gender information so that a general and robust bone age prediction system can be developed in case of privacy concerns in providing the gender information. Hence, a general bone age prediction that completely relies on X-ray images is more preferable to mitigate this issue.

Therefore, the main contributions of this paper can be summarized as follow:

1. Integration of the spatial-wise attention mechanism that aims to allocate more weights towards the optimal regions of interest.
2. Better distribution of training, validation, and testing data, where they are distributed according to the ratio of 8:1:1, respectively.
3. Extensive comparison for the data normalization stage that covers state-of-the-art deep learning architectures for segmentation and point of interest localization.
4. A more robust bone age assessment system is produced without utilizing the gender information. This step is taken to support patient’s privacy issues where some of them are reluctant to disclose their gender information.

## 3. Related Works

Over the past years, there are plentiful published works on the development of automated bone age assessment systems which can be broadly categorized into conventional machine learning and deep learning approaches. Generally, the TW approach has been more widely adopted compared to the GP approach due to its modular structure that allows features to be extracted from selected regions of interest. Hence, the conventional machine learning approach explores various handcrafted features from the specific ROIs to train the classifier or regressor to predict the bone age. According to Dallora et al. [31], the most popular conventional machine learning algorithms used to predict the bone age are artificial neural networks (ANN), and support vector machine (SVM) regressors. In [32], an ensemble technique has been proposed to extract a set of features from each finger’s joint which is later passed to a linear regressor to predict the final bone age. Instead of focusing on the finger’s features, the work in [33] has focused on utilizing features from carpals regions to estimate the bone age. On the other hand, methods in [34,35] have employed ANN regressor to determine the bone age.

Contrary to the previous works, Tang et al. [34] have utilized MRI instead of X-ray images. They have proposed four different input features, which are weight, height, TW age score, and intensity of the MRI signal as an input to train their ANN regressor. A simplified feature set of bone scores from different stages has been used in [35] to train their ANN model. Kashif et al. [36] then implemented various key-points features from SIFT, SURF, BRIEF, BRISK, and FREAK to further improve the ANN complexity. They later applied SVM with a polynomial kernel to further improve the estimation of the bone age. Conversely, a systematic approach by Sheshasaayee and Jasmine [37] have used conflated Gabor, local binary pattern, and color histogram features to train their SVM regressor to predict the bone age more efficiently. In addition to that, Simu and Lal [38] have tested three different features that comprise of texture data, a histogram of oriented gradients (HOG), and a bag of features (BoF), which are extracted from the phalanges bone regions as an input to Random Forest predictor. Henceforth, selecting the right and suitable features to train the predictor or regressor is a crucial task in the conventional machine learning approach.

Due to the need of optimizing the feature selection, the deep learning approach has been adopted by many researchers to alleviate the drawbacks of the handcrafted features. The earliest automated system that has applied deep learning architecture was designed by Spampinato et al. [28] through their BoNet architecture. The model utilizes a straightforward CNN with five convolutional layers. The proposed BoNet architecture outperformed other existing deep learning models such as OverFeat, GoogLeNet, and OxfordNet with the best MAE of 9.5 months. Similarly, Lee et al. [29] have explored several CNN architectures, in which GoogLeNet has returned the highest efficiency in estimating the bone age. In addition, they have also employed a pre-processing pipeline to standardize the input images and eliminate the unnecessary noise to enhance the model’s accuracy, which is in contrast to BoNet that does not perform any pre-processing steps.

In general, training a deep learning model requires a vast amount of images so that the model will not suffer from underfitting and overfitting problems. Therefore, the work in [39] has utilized pre-trained VGGNet weights and data augmentation to mitigate their problem of limited training data. They have employed average-based model fusion among the CNNs to finally assess the bone age. Moreover, they have defined five ROIs that cover carpal and phalanges regions based on the domain knowledge by the radiologists. Moreover, a similar ROIs division has been introduced in [40], in which they have argued that obtaining specific features will further improve the regressor performance in predicting the bone age. Then, each of these regions has been trained using three different deep learning models, namely DenseNet-121, Inception-V3, and InceptionResnet-V2, which will be the input to a Random Forest regressor. Nevertheless, Tang et al. [41] have employed the transfer learning technique by utilizing RSNA dataset to first pretrained their TjNet architecture which has been constructed using CNNs.

Meanwhile, the work in [1] has utilized two deep learning models, namely U-Net and VGGNet to provide an end-to-end network that automatically streamlined the bone age prediction. The U-Net is tasked to segment the hand X-ray images, while the VGGNet is used to detect the key-points and estimate the bone age. The work by Ren et al. [2] has introduced a simple attention mechanism to produce coarse and fine attention maps applied to Inception-V3 architecture. Likewise, Chen et al. [42] have extracted three attention maps derived from the whole hand region, first-most, and second-most discriminative regions, respectively also using the Inception-V3 model. The work in [43] has provided an end-to-end system to segment the X-ray images and predict the bone age. The hand region has been segmented using the Mask R-CNN, while the bone age estimation is performed through a simple residual attention network.

Most of the recent bone age prediction researches are still relying on standard CNN architectures. According to the work by Reddy et al. [44], they have designed a fully automated bone age prediction focusing only on the index finger rather than utilizing the whole hand X-ray images. They have argued that this reduced region of interest approach can alleviate the problem of a small-scale dataset that they have encountered during the development process. Albeit a small test dataset, their method has returned a relatively good prediction on the bone age just by relying on a single index finger. Similarly, the work by Marouf et al. [45] has also implemented CNNs to provide an end-to-end system to predict the bone age. They have utilized the powerfulness of CNNs to predict bone age with the help of gender information with a mean absolute difference (MAD) of 0.5 years, where the gender classification used is less accurate with just 79.60% classification accuracy. In another study, Pan et al. [46] have explored several CNN architectures that include Inception-V3, Xception, and InceptionResNet-V2. Their best result is obtained by using InceptionResNet-V2 that has returned the highest MAE as compared to the other baseline models. Moreover, they have highlighted that their proposed method can be used to alleviate the annotation burden so that more training data can be obtained. They have also pointed out that the transfer learning technique is able to improve the accuracy of the training process for the InceptionResNet-V2 with a lesser number of epochs. Lastly, Hao and Li [47] constructed an end-to-end approach through EfficientNet regressor to predict the bone age which has been trained using the RSNA dataset. They have utilized the B3 model that uses 12,757,296 optimal parameters distribution between the number of channels and network depth through network architectural search approach. Table 1 describes the reviewed deep learning models for bone age prediction.

## 4. Methods

**Figure 3:**
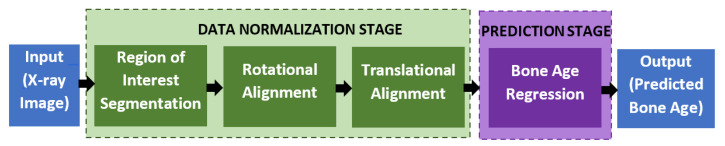
The workflow of the proposed automated assessment of the bone age.

The proposed automated bone age assessment system will involve four-stage modules, which are the region of interest segmentation, rotational alignment, translational alignment, and bone age regression. Figure 3 shows the full workflow of the system, starting from the X-ray input until the predicted output of the bone age assessment. The first three modules perform a data normalization task that transforms the input image into a standardized form so that the regressor networks can better predict the bone age with high accuracy. The normalization procedures follow the same approach as used in [30]. Furthermore, various other deep learning architectures have been tested to find and validate the optimal network architectures for the data normalization, which is discussed in the Experiment and Discussion section.

### 4.1. Data Normalization

Let the X-ray input image be represented by *I*, then, the masked region of interest module will produce a set of $I_{k,masked}=f_{masked}\left(I_{k}\right),k=1,2,\ldots ,T_{I}$, where $T_{I}$ is the total number of X-ray images. $I_{masked}$ is the segmented hand region that removes all the background information such as medical devices, reference plate, reference numbers, unrelated body parts, and many more. Several state-of-the-art segmentation architectures have been tested and validated to find the best hand segmented region that includes Stacked U-Net [48], PSPNet [49], DenseDeepLab V2 [50], FCN [51], DeepLab V1 [52], DeepLab V2 [53], DenseDeepLab V3+ [50], SegNet [54], FC DenseNet [55], U-Net [56], and DeepLab V3+ [57]. During this stage, the hand region will be extracted using the deep learning segmentation technique so that any background information can be excluded for future processing. The goal is to produce a masked image $I_{k,masked}$ by subtracting $I_{k,mask}$ from $I_{k}$. The mask label is set to high for the foreground region, which is the hand region, and vice versa. $I_{k,mask}$ for each method is trained by using data division of 4:1 between training and testing datasets. The X-ray images will be resized to fit the input requirements by each of the methods, where the most popular input size is 224 × 224, which are used by FCN, SegNet, and FC DenseNet. Moreover, all variants of DeepLab methods use 321 × 321 input images, while the largest input size is 512 × 512 required by stacked U-Net. Each of the methods will be trained until convergence by using a fixed learning rate of 0.0001 through Adam optimizer update. Once each of the methods has been trained until the parameters are optimized, the segmentation test is performed to find the best deep learning architecture that suits the problem of hand X-ray segmentation.

**Figure 4:**
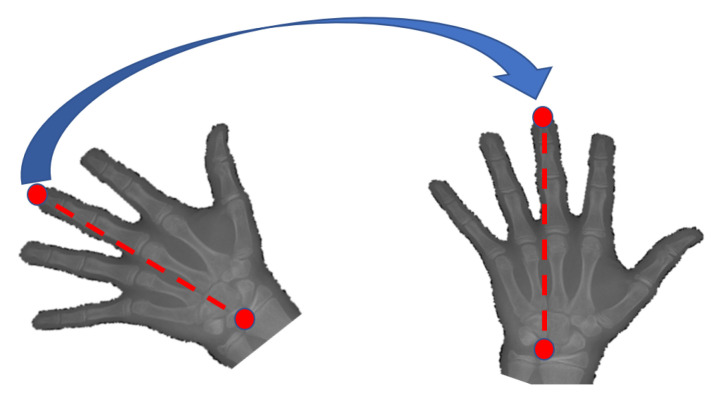
Key-points alignment that rotates the segmented hand region such that the tip of the middle finger and the lower end of the carpal region form a straight vertical line.

The segmented hand region is then passed to a rotational alignment module that adjusts the rotational placement of the hand using a key-points detector. Four points of interest will be observed which are the tip of the thumb, the tip of the middle finger, the tip of the pinkie finger, and the lower end of the carpal region. These four points are selected since they represent the outer points of our region of interest, which is the palm region. Several recent key-points detectors that are based on convolutional neural networks have been tested and validated that include ResNet-50 [58], GoogleNet [59], ShuffleNet V1 [60], ShuffleNet V2 [61], MobileNet V1 [62], MobileNet V2 [63], MobileNet V3 [64], SqueezeNet [65], LightCovidNet [8], Xception [66], DenseNet [67], and SPPCovidNet [7]. The coordinates for these four key-points are denoted as $P=\{p_{1},p_{2},p_{3},p_{4}\}$, where $p_{i}$ is the Cartesian coordinate with respect to the origin that is set at the top-left point of the image. Therefore, each of the key-point detectors will be trained also using Adam optimizer backpropagation method to optimally locate the P set. A similar data division approach is used between training and testing datasets through a ratio of 4:1. Once the key-points have been detected, a set of $I_{rotate}$ images is then obtained by aligning the angle between the tip of the middle finger and the lower end of the carpal region such that these two points will create a straight vertical line as shown in Figure 4. Even though there are four key-points identified using the key-point detector, only two points are needed to calculate the rotational alignment angle as follows:

(1) $$\begin{array}{c} \theta =\cos^{-1}\left(\frac{p_{2}\left(y\right)-p_{4}\left(y\right)}{\sqrt{{\left(p_{2}\left(y\right)-p_{4}\left(y\right)\right)}^{2}+{\left(p_{2}\left(x\right)-p_{4}\left(x\right)\right)}^{2}}}\right) \end{array}$$

where (x,y) refers to the Cartesian coordinate of point *p*. The two selected key-points are the tip of the middle finger and the lower end of the carpal region. The final normalization module, which performs translational alignment, will place the segmented and rotated hand image in the middle of the image such that the center point of metacarpal coincides with the center point of the image to produce a set of normalized image $I_{normed}$. These processed images are then passed to the bone regression module to predict the bone age using the proposed AXNet. For each normalization step, the greedy approach is used by selecting the best method output to be the input for the next step. Therefore, the output of the best deep learning segmentation method is used as an input for the rotational alignment test. The hand segmented image will be extracted first using the most accurate segmentation method which will be the input for the key-points regressor test. Therefore, the best output of the rotational alignment indicates the best normalization method since translational alignment is just deterministically placed on the hand region in the middle of the image.

(2) $$I_{k,normed}=f_{translational}\left(f_{rotate}\left(f_{masked}\left(I_{k}\right)\right)\right)$$

### 4.2. Bone Age Regression

**Figure 5:**
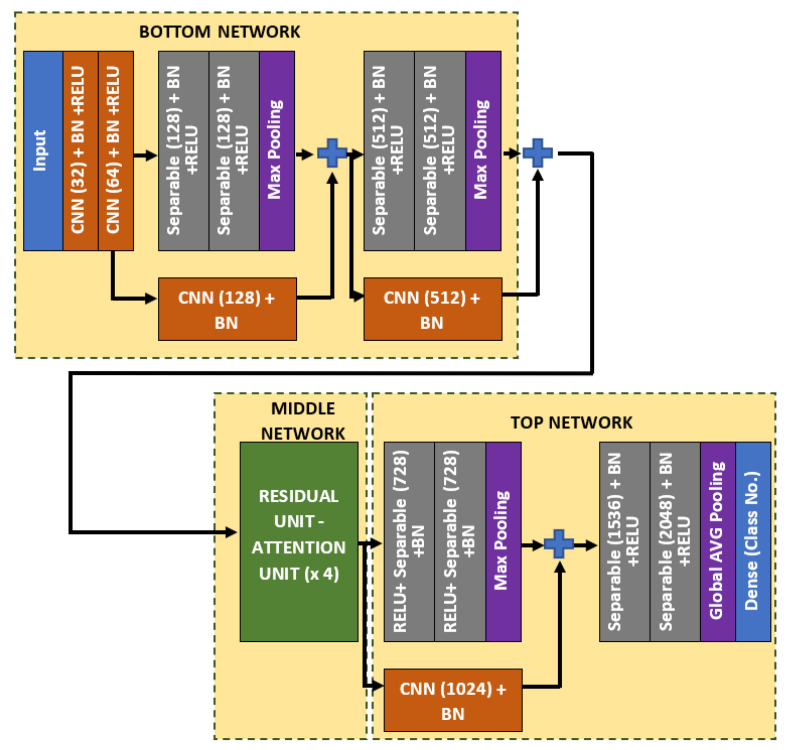
The full architecture of the proposed Attention-Xception Network.

In this work, a spatial-based attention mechanism is embedded in the bone age regressor network. The purpose of this attention network is to emphasize more weightage on the region of interests, such that the age can be better differentiated. The regressor network, which is remodeled from Xception architecture is named as Attention-Xception Network (AXNet). The full AXNet consists of three parts, which are the bottom, middle, and top networks as shown in Figure 5. In the bottom network, two residual skip connections are added right after the max-pooling operators, which will reduce the effective size of the feature maps, but produce a bigger set of channel layers. Generally, separable convolution, which is a factorized version of the standard convolution is utilized as the network building block to reduce the memory usage, except for the initial stage convolutions and skip connection convolutions. The middle network consists of four consecutive pairs of residual and attention units, followed by a single skip connection unit, which will down-pool the feature map size from 12 × 12 × 2048 to just 1 × 1 × 2048. Global average pooling is used instead of the maximum pooling scheme, which will be regressed to a single node prediction.

**Figure 6:**
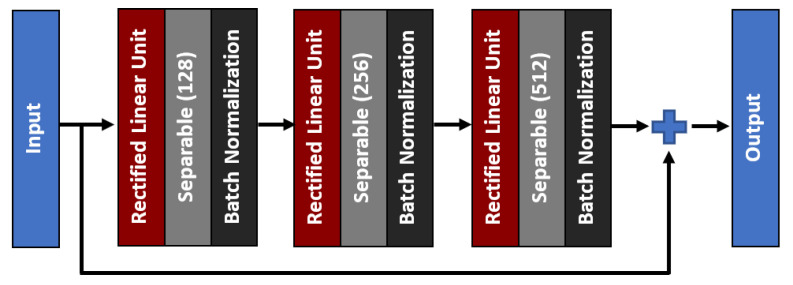
The architecture of a three-layer residual skip connection unit.

**Figure 7:**
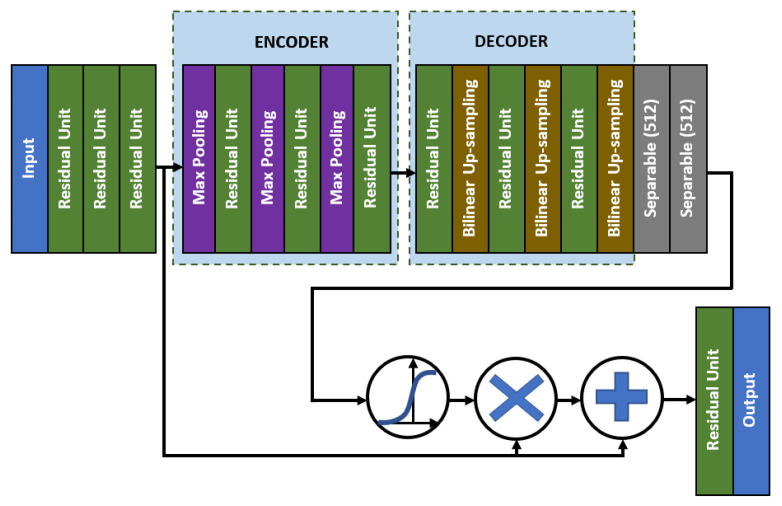
The full architecture of the attention mechanism in AXNet.

As for the middle network, the residual skip connection will be composed of a three-set of separable convolution layers with an expanding number of filters from 128, 256, to 512. The kernel size of the filter follows inverted bottleneck architecture with the first and the third layers composed of 1 × 1 kernel, while the bottleneck layer has a larger kernel with a 3 × 3 filter. The residual unit architecture is shown in Figure 6, whereby the skip connection is passed directly from the input to the last layer output, which is then combined using an addition operator. For the attention unit, there will be two paths, the primary and the secondary. The primary path will undergo three layers of residual unit operation, which will be later masked by the secondary path. The secondary path will create the mask to mark the regions of interest through an encoder-decoder network using three layers of downsample and upsample operations. The purpose of this encoder-decoder scheme is to find the best latent feature representation, which will act as the filter so that more weightage can be allocated to particular regions. The secondary path is then passed to a sigmoid activation function to create a mask with weights in the range of [0, 1]. The mask is then combined with the primary path before finishing the attention mechanism with a residual unit. Figure 7 shows the full architecture of the attention mechanism used in AXNet. The detailed configuration of the AXNet is shown in Table 2. The regressor is then trained to predict the bone age using the input from X-ray images, which have been divided into train, validation, and test datasets according to the ratio of 8:1:1.

## 5. Results and Discussion

This section describes the training configurations and evaluation metrics used for performance analysis. The results of the best architecture for hand masked segmentation, key-points detection, and bone age regression are discussed individually according to their sequence of application. The proposed AXNet will be benchmarked with state-of-the-art deep learning models.

### 5.1. Dataset

There are a total of 14,236 X-ray images that have been extracted from Pediatric Bone Age Machine Learning Challenge [68], which were collected by the Radiological Society of North America (RSNA). The original dataset composition consists of 12,611 training images, 1425 validation images, and 200 testing images. However, the small number of testing datasets allows the overfitting of a certain network that focuses only on that 200 images. Therefore, for a better dataset distribution, we have divided the whole 14,236 images randomly into training, validation, and testing datasets according to the ratio of 8:1:1, respectively. The X-ray images were taken primarily from two children’s hospitals in the United State of America; Children’s Hospital Colorado and Lucile Packard Children’s Hospital. Six medical practitioners have been tasked to annotate the age of each X-ray image, where the final decision is based on the weighted average of each annotator. Any slight difference among the annotators can be attributed to the contrast and brightness differences among the images as well as posed variation of the test subject’s hand. There are various X-ray machines used to collect the data that vary from the lowest resolution of 800 × 1011 pixels up to the highest resolution of 2460 × 2970 pixels. Originally, the images were stored in DICOM format, which is then converted to Portable Network Graphic format with 8-bit image depth. Out of 14,236 images, 6530 of them were captured from female test subjects, while 7706 of them were captured from male test subjects. In this work, gender information is omitted because of data privacy issues, where this research aims to produce a robust assessment method regardless of gender. The range of the test subjects’ age is from 1 to 228 months, where normal people will stop growing after that and hence bone age prediction is not valid anymore.

### 5.2. Training Configurations

All of the experiments have been coded on the Python platform with Keras front-end and Tensorflow back-end, which is trained parallelly using NVIDIA RTX 2080 Ti graphics processing unit (GPU). Adam optimizer with a fixed learning rate of 0.0001 has been utilized to train the networks for the hand masked segmentation and key-points detection. Meanwhile, the training processes have been set to employ binary cross-entropy loss function for the hand masked segmentation and mean squared error loss function for the key-points detection. Similarly, the proposed AXNet and its benchmarked models have been trained using Adam optimizer for 150 epochs. A batch size of 16 images is used during the training phase, which is limited by the memory of the NVIDIA RTX 2080 Ti. Data augmentation is also used to supplement the training data using rotation, zooming, translation, shearing and flipping functions. The rotation angle is set to a step size of 20${}^{\circ }$, while zoom range, shift factor, and shear factor are set to the ratio of 0.15, 0.2, and 0.15, respectively. The input size requirement for the proposed regressor is 288 × 288. The loss function of mean squared error is used during the learning process, where the learning rate, Lr will follow a piece-wise scheme as shown below:

(3) $$Lr=\left\{\begin{array}{c} 0.0010,epoch<50\\ 0.0005,50\leq epoch<100\\ 0.0001,epoch\geq 100 \end{array}\right.$$

The goal of this piece-wise function is to reduce the learning rate value for every 50 epochs and the learning momentum is fixed to 0.9 for all epochs. The initial learning rate is set to the standard Adam optimizer learning rate, which is 0.001, where it is reduced by half, 0.0005 between 50 to 100 epochs. Then, during the final stage, the learning rate is reduced significantly as the training error becomes small, which will help to reduce fluctuation in the parameter updates.

### 5.3. Performance Metrics

Five evaluation metrics are used to validate the proposed method that includes mean accuracy $\left(\bar{Acc}\right)$, mean intersection over union (IoU), Hausdorff distance ($H_{Dist}$), mean absolute error (MAE), mean squared error (MSE), and total number of parameters. The hand masked segmentation results are evaluated using $\bar{Acc}$, IoU, and total parameters. On the other hand, both key-points detector and bone age regressor performance are validated using MAE, MSE, and total parameters. The $\bar{Acc}$ is defined as the ratio of the correctly classified pixels and the total number of tested samples as shown in the following equation.

(4) $$\bar{Acc}=\frac{\sum_{i=0}^{N_{test}}\frac{TP_{i}+TN_{i}}{TP_{i}+TN_{i}+FP_{i}+FN_{i}}}{N_{test}}$$

where $TP_{i}$ is the true positive, $TN_{i}$ is the true negative, $FP_{i}$ is the false positive, $FN_{i}$ is the false negative detection, and $N_{test}$ is the total number of the tested samples. Whereas, IoU measures the ratio of the overlapping region between the segmented images, $S_{segment}$, and its ground truth images, $S_{gt}$.

(5) $$IoU=\frac{\sum_{i=0}^{N_{test}\frac{S_{segment,i\cup S_{gt,i}}}{S_{segment,i}\cap S_{gt,i}}}}{N_{test}}$$

For $H_{Dist}$, the metric only concerns the hand segmented regions, where it compares the distance in pixels between the segmented output and the ground truth data. Let us denote the two points’ set contours that surround the ground truth segmented regions ($R_{C}$) and segmented regions from the deep learning ($R_{D}$) as *C* and *D*, respectively. A one-directional $H_{Dist}^{single}$ is formulated as follows:

(6) $$\begin{array}{c} H_{Dist}^{single}\left(C,D\right)=\max_{c\in C}\left\{\underset{d\in D}{\sup}||c-{d||}_{2}\right\} \end{array}$$

Then, the final bi-directional $H_{Dist}$ is calculated as follows:

(7) $$\begin{array}{c} H_{Dist}\left(C,D\right)=\max\left\{H_{dist}^{single}\left(C,D\right),H_{dist}^{single}\left(D,C\right)\right\} \end{array}$$

Meanwhile, MAE measures the average of the absolute difference between the annotated values, $B_{gt}$, and the predicted values, $B_{predict}$. Similarly, MSE refers to the average of the squared difference between $B_{gt}$ and $B_{predict}$. Lastly, the total parameters refer to the total number of memory registers used to construct the deep learning model, where a deep and wider model will generally utilize a large number of total parameters and vice versa. It can be simplified as the number of network units that can be trained and optimized, which include the variables for convolution kernels, biases, dense connection, and batch normalization. Thus, a network with a big total number of parameters indicates that it requires more memory storage to store all its variables’ data. As consequence, a larger network will have a smaller number of batch sizes during the training phase due to the limitation in GPU memory size. In general, a deep learning network requires at least 64 batch sizes for optimal training as proven in [69]. On the other hand, a network with a low total number of parameters is more preferable for mobile applications.

(8) $$MAE=\frac{\sum_{i=0}^{N_{test}}\left|B_{gt,i}-B_{predict,i}\right|}{N_{test}}$$

(9) $$MSE=\frac{\sum_{i=0}^{N_{test}}{\left(B_{gt,i}-B_{predict,i}\right)}^{2}}{N_{test}}$$

### 5.4. Results and Discussion: Hand Masked Segmentation

Table 3 lists out the performance measures of the hand masked segmentation methods that have been tested and validated to extract the hand region foreground. Based on the results, DeepLab V3+ produces the best segmentation performance with the highest $\bar{Acc}$ and IoU of 97.826% and 0.94778, respectively. These significant results can be attributed to the inherent capability of DeepLab V3+ that utilizes parallel atrous separable convolutions that better capture the features of various scales. This is because the dataset was captured in various scales form where the distance between the hand and the X-ray machine is not fixed as it depends on the machine configurations. Since the images were captured from several machines from two different children’s hospitals, there will be slight variations in the scale setup. The experimental results also indicate that U-Net, FC DenseNet, and SegNet, which have utilized a symmetrical encoder-decoder configuration, produce slightly less accurate $\bar{Acc}$ and IoU performances but using less number of parameters. These three models have utilized several layers of upsampling layers that manage to reconstruct back the latent variable from the bottleneck layer.

Moreover, it is interesting to note that even though DeepLab V3+ requires more memory storage compared to its closest competitor with a total number of parameters of 41,253,888, the memory requirement is still within an acceptable range compared to the DeepLab V2 and FCN architectures. In addition to that, the $H_{Dist}$ for DeepLab V3+ is 3.791 pixels compared to the second best model, U-Net with $H_{Dist}$ equal to 2.897. Despite the fact that it is higher, it is still considered as a very small error, where both $\bar{Acc}$ and IoU play a more important role in selecting the best segmentation method. This is because a better-segmented hand region is more crucial for future processing of bone age assessment. A slightly higher $H_{Dist}$ indicates that there are a few outputs of extracted hand regions that have been over-segmented or under-segmented, which makes it slightly less accurate. However, the role of $\bar{Acc}$ and IoU are more important because these two metrics treat the contribution of every pixel equally, whereas $H_{Dist}$ only concerns on certain pixels that produce the maximum distance between the model output and the ground truth mask. In addition to that, the largest model, which is FCN, has the lowest $H_{Dist}$ of 2.747 but a relatively less accurate $\bar{Acc}$ and IoU performance. These inaccuracies can be attributed to more over-segmented or under-segmented regions with no extreme distance between the model and the ground truth, where $H_{Dist}$ only considers a single point of the maximum distance.

In contrast, DenseDeepLab V3+ has produced a weak segmentation performance even though more feed-forward layers have been applied compared to the original DeepLab V3+. This is in line with the nature of this hand masked segmentation task that focuses on gray channel imaging for two-class classification, where the advantage of having more feed-forwarded layers is limited. Similarly, PSPNet, that utilizes a set of pyramid pooling layers, has also performed relatively worse, whereby the advantage of its multi-scale pooling is diminished because of its limited upsample layers. PSPNet implements an asymmetrical encoder-decoder scheme with just overlineAcc and IoU of 97.084% and 0.93094, respectively. In addition to that, the lightest model, stacked U-Net with just 3 million parameters performed the worst with $\bar{Acc}$ and IoU of just 95.330% and 0.88967, respectively. The low performance of stacked U-Net compared to the original U-Net can be attributed to the repeated units of shallow encoder-decoder networks that cannot be trained well given the low contrast in the X-ray imaging. It is important to use a deeper and wider encoder-decoder network to cover and detect even a small change, especially for the hand masked application. Hence, DeepLab V3+ has been selected as the best deep learning model for the hand masked segmentation, which is then passed to the rotational alignment module.

### 5.5. Results and Discussion: Key-Points Detection

Following the hand masked segmentation output, the key-points detector is then executed as part of the data normalization process to properly align the images in an upright position. Similar to the hand masked segmentation, several state-of-the-art key-point detectors have been compared to find the best model to optimally rotate the segmented hand images. The experimental results of the key point detectors are shown in Table 4. It can be seen that the MobileNet V1 model has returned the smallest MAE and MSE of just 0.0356 pixels and 0.01409 pixels^2^, respectively followed by DenseNet-264 and SPPCovidNet. It is worth noting that the usage of depthwise-pointwise convolution in the MobileNet V1 has produced excellent four key-points detection even though it is the lightest model with slightly more than 3 million parameters. Contrary to the second-best model, DenseNet-264 requires a large total number of parameters of 10 times more than MobileNet V1, albeit producing slightly lower performance. The large size of DenseNet-264 architecture is caused by the accumulation of repeated feed-forward layers that are concatenated together, which are then added on top of the respective existing channel information.

Moreover, the other state-of-the-art deep learning models that employed separable convolution schemes, such as Xception-41, Xception-71, MobileNet V2, MobileNet V3, ShuffleNet V1, and ShuffleNet V2, produce slightly less accurate MAE compared to the top three models. In both Xception-71 and Xception-41 models, there is an additional of three-layer residual connection in their networks, which allows it to produce better performance compared to the early models such as GoogleNet. By having a residual skip connection, the network reduces the possibility of diminishing gradient problems for training a deep learning network. Thus, the early layers will also play role in learning the best feature representation instead of just the latter layers. Moreover, the original residual skip connection model, ResNet produces the worst performance with relatively high MAE and MSE of 0.12444 pixels and 0.04091 pixels^2^, respectively. This is because the original version is just using a simple two-layer skip connection without utilizing any shuffle or deeper channel learning. Therefore, the three-layers of the residual module with a bigger kernel in the middle layer produces better key-points detection compared to just a simple skip connection scheme. Henceforth, the results show the capability of both Xception-71 and Xception-41 models that compose linear stacks of the depthwise separable convolution layers with residual connections is more efficient and accurate. Despite the low MAE, both Xception-71 and Xception-41 require big memory usage with total parameters of 35,640,704 and 20,877,872, respectively.

**Figure 8:**
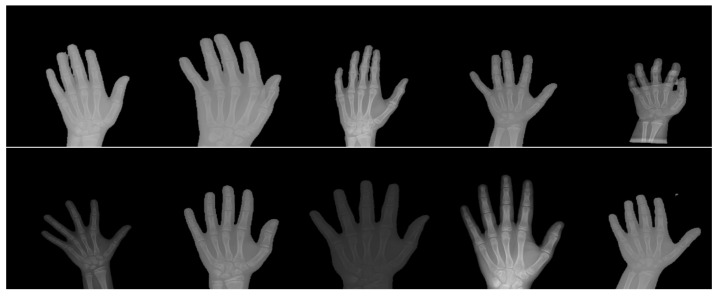
Some samples of the X-ray image that have been normalized by the three normalization modules.

Meanwhile, ShuffleNet achieved the fourth lowest MAE among the state-of-the-art deep learning models, where the group and shuffle layers do not work well in extracting the key-points feature. It is important to consider the case of similar phalanx regions, which will be shuffled in the case of ShuffleNet and hence produces a low accuracy of key-points detection. This reasoning is also supported by the poor performance of the ShuffleNet V2 model where the features are shuffled in two paths at the beginning of the shuffle module. On the other hand, as the lightest model with just 739,600 parameters, SqueezeNet has only returned an MAE and MSE of 0.06448 pixels and 0.02091 pixels^2^, respectively. It performs poorly compared to the SPPCovidNet where the error performance is much lower but with a slightly bigger network. The basis of the SqueezNet and SPPCovidNet is similar but SPPCovidNet has an additional module of simplified spatial pyramid pooling that allows the network to better learn multi-scale information. The improvement of just adding the module is significant where the MAE is reduced from 0.06448 to 0.04138, which is a reduction of 36% in error. Therefore, a multi-scale unit is an important module for the case of finding the key points of interest in the hand X-ray images, whereby the multi-scale information is important due to the variations in the way of capturing the images. Based on overall performance, MobileNet V1 is chosen as the best model for the key-points detection due to the lowest MAE and MSE values as well as the lightweight model with less than 4 million parameters. Figure 8 shows some samples of the normalized X-ray image that will be fed to the bone age regressor network.

### 5.6. Results and Discussion: Bone Age Assessment

Table 5 provides the experimental results of the bone age prediction for the AXNet and its benchmarked state-of-the-art models. The proposed AXNet with four layers of spatial-attention mechanism has produced the lowest MAE and MSE with 7.6996 months and 108.869 months^2^, respectively. The second best performance is achieved by the method by Zulkifley et al. [30] with MAE and MSE of 8.200 months and 121.902 months^2^, followed by the original Xception-41 with MAE and MSE of 8.357 months and 121.155 months^2^, respectively. Thus, the modified spatial-attention mechanism has improved the prediction performance by lowering the MAE and MSE by 7.86% and 10.14%, respectively while increasing the total number of parameters by a small increment of 0.82%. The performance increment can be attributed to better emphasization of the regions of interest through the attention mechanism. As proven by the TW approach, several regions, such as phalanx areas, are more important in deciding the bone growth compared to other regions. Moreover, there are four layers of attention mechanism have been applied, which can capture a wider range of unique features of the particular ages. In addition, the second-best method has also applied the Xception architecture as the base network, but without any attention mechanism that is able to emphasize more weights on the regions of interest. It is interesting to note that the same architecture will perform differently using different data normalization methods, where Xception-41 by Chollet et al. [66] produces a better MSE value but a worse MAE value.

In addition to that, some early architectures of CNN such as Inception V3, ResNet, and DenseNet have performed better compared to the newer models. All these three models have applied skip connection mechanism without applying any factorized separable convolution, where the first two architectures use an addition operator to merge the features, while the last architecture uses a concatenate operator. Their performances are relatively similar in terms of MAE, but ResNet has a noticeable high MSE compared to the other two architectures. This anomaly is caused by large wrong predictions in certain test images for ResNet, while the prediction skew for the Inception V3 and DenseNet are much smaller. In addition to that, Pan et al. [46] have utilized InceptionResNet V2 in designing their regressor network. Their regressor performs better in terms of MAE and MSE compared to the newer version of Inception V3, but it requires 3 times more total number of parameters. This is because the encoder part utilizes the ResNet architecture with a deeper network compared to the original Inception network. The MSE improvement is significant from 191.696 months^2^ to 152.328 months^2^, which is a reduction of more than 20%. In addition to that, InceptionResNet also implements a variable size kernel coupled with residual connection, which makes it perform better compared to the Inception V3.

On the other hand, the work by Lee et al. [29] utilizes GoogleNet or Inception V1 architecture, which has produced a much worse MAE and MSE of just 10.972 months and 220.759 months^2^, respectively, compared to the newer version of the Inception architectures. This is because the original Inception is just a 22-layer network architecture without employing any residual connection. Due to its shallower network, it cannot extract the comprehensive features set compared to the newer Inception architectures that have more than 50 network layers. A similar trend can be observed in the work by Iglovikov et al. [1] that utilizes VGGG-16 architecture compared to the VGG-19 architecture. VGG-19 bone age regressor produces a better performance with 5.24% reduction in MAE and 11.98% reduction in MSE compared to the work by Iglovikov et al., whereby VGG-19 utilizes an additional three convolution layers compared to the VGG-16. However, the biggest network with 95,116,161 parameters by Spampinato et al. [28] performs relatively moderate with MAE and MSE values of 10.972 months and 220.759 months^2^, respectively. They have modified the Overfeat architecture to include a deformation layer between the last two convolution layers. This model has a large total number of parameters because of the implementation of big kernel sizes in the first two convolution layers with 7 × 7 and 5 × 5 kernels. This is in contrast to the recent trend in deep learning architecture design that utilizes either 3 × 3 or 1 × 1 convolution kernels, where a bigger feature map size can be achieved through dilation convolution, while maintaining a relatively low total number of parameters.

On the other hand, the best mobile-based CNN architecture is produced by MobileNet V1 with MAE and MSE of 10.886 months and 190.349 months^2^, respectively. It is crucial to note that most of the lightweight models do not perform well compared to the models with more than 15 million parameters. Since the range of the regression output is from 1 to 228 months, the prediction network requires a more complex mapping from the X-ray image to a single node regression output. However, a straightforward network of convolution stacking with a large number of parameters without any skip connection and attention mechanism such as VGG-19 will not perform well because of the low cross-mapping between the features. Even more, in the case of the X-ray image, the information contained in the image is limited with just gray channel data with low contrast conditions. Therefore, a well-designed network that utilizes cross-layer information is important for the case of bone age prediction using X-ray images. The lowest MAE and MSE are reported by ShuffleNet V1 with 15.728 months and 372.575 months^2^, respectively, which are more than twice the errors reported by AXNet. In addition to that, ShuffleNet V2 also does not fare well with the AXNet performance. High errors in predicting the bone age for these two models can be attributed to the application of channel shuffle. In fact, more channels are shuffled in ShuffleNet V1 compared to ShuffleNet V2 with just two-channel paths. The shuffling process allows the network to learn from different inputs with the aim to limit feature generalization of the whole network. For the case of bone age regression, a unified generalization that covers wider feature maps works better as proven by the networks with a big total number of parameters. Hence, AXNet with more than 15 million parameters, equipped with attention mechanisms and residual units performs the best compared to the other state-of-the-art CNN models.

### 5.7. Results and Discussion: Ablation Study

There are two main novelties that have been pursued in this study, which are optimal data normalization and attention network. Therefore, Table 6 shows the contribution of each module in improving the AXNet performance. Without an optimal data normalization module, AXNet performance in assessing the bone age has reduced from the best MAE of 7.699 months to 8.219 months and the best MSE of 108.869 months^2^ to 119.240 months^2^. Without a data normalization module, the input images are not transformed to the standardized pose, which has forced the regressor network to learn more non-discriminative information that can distinguish the age. With an optimal data normalization module, the input to the AXNet has been transformed into a standardized pose that allows the regressor network to better learn the distinguishing bone features of different ages. In addition to that, the performance of AXNet without the attention unit is more affected compared to the AXNet without the data normalization module. In fact, the MAE has reduced by a larger factor from 7.699 months to 8.357 months. Furthermore, the MSE also has reduced from 108.869 months^2^ to 121.155 months^2^, which is a performance reduction of 10.14%. Therefore, the role of the attention unit is very crucial in assessing the bone age accurately. The addition of this module allows the network to better distinguish the features that come from the selected regions of interest such as phalanges and carpal regions compared to the other less important regions. Hence, by emphasizing these regions through the attention module, the network has learned the unique features in predicting the age of the subject. This reasoning is in line with the TW approach that derives its scores from several small regions of interest.

## 6. Conclusions

This paper has proposed an automated bone age assessment model, AXNet, that can serve as a second opinion for radiologists in assessing bone growth anomaly. AXNet can also serve as the confirmation tool that helps inexperienced radiologists, especially in rural areas where the expert is not available. In addition to that, due to the subjective nature of assessing the bone age manually, this work can also supplement the radiologist’s decision-making in determining the bone age of the patients. The proposed AXNet has utilized a set of three image normalization modules that convert the original X-ray images into a standardized form for better training procedures of the regressor network. The three modules will first segment the hand region, which is then rotated until the key-points between the middle fingertip and the lower end of the carpal region form a straight vertical line, before translationally aligned to be positioned in the middle of the image. According to the experimental results, DeepLab V3 has been selected as the best model for the hand masked segmentation task that returned the highest $\bar{Acc}$ and IoU values. Meanwhile, this study has also revealed the capability of MobileNet V1 for MAE and MSE in detecting the key-points detection for rotational alignment module with the lowest MAE and MSE as well as a considerably lightweight model of just 3,237,064 parameters. The proposed AXNet has also managed to achieve the smallest MAE and MSE for the bone age prediction with 7.6996 months and 108.869 months^2^, respectively, without applying any gender information. The results indicate that the implementation of the attention mechanism has improved the network capability by emphasizing more weights on selected regions of interest as produced by the attention maps. Moreover, AXNet has also utilized a 3-layer residual separable convolution that reduces the probability of diminishing gradient problems during the training process. Finally, AXNet computational burden is also moderate with a total number of 21,035,545 parameters.

## 7. Limitation and Future Work

The current limitation of this work is the lack of multi-scale capability, which limits the model performance to generalize well for man and woman subjects. Spatial pyramid pooling or atrous pyramid pooling can be embedded into the network to extract multi-scale features that can learn better features for genderless bone age regressor. However, the number of parallel scales needs to be optimally selected because of the increased computational burden. Moreover, the placement of the multi-scale module needs to be carefully examined, whereby embedding it at the bottom layer of the network will limit the number of parallel scales due to the smaller available feature map size. In addition to that, this work does not apply any size normalization to adjust the hand region size before it is passed to the regressor network. Intuitively, the bone age can be better assessed if the size of the extracted regions is standardized, where the attention network can better focus on the standardized regions of interest. Size normalization can be performed by finding the outer contour of the hand region and scaling it down/up to the selected representation ratio.

## Figures and Tables

**Table 1:** Overview of the deep learning development for bone age prediction.

Method	Model	Strength	Weakness
Spampinato et al. [28]	BoNet	-Uses a simple CNN architecture with only five convolutional layers.	-Does not employ any pre-processing pipeline. -Does not embed residual or feed-forward connections.
Lee et al. [29]	GoogLeNet	-Applies pre-processing pipeline to produce standardized images.	-The experiment has included radiologists report for training process, which will give advantage for bone age prediction performance.
Zhou et al. [39]	CNNs	-Uses various ROIs based on domain knowledge to increase model accuracy.	-The model is not trained until convergence because the model accuracy is still increasing before the training has been ended.
Wibisono and Mursanto [40]	DenseNet and InceptionResNet V2	-The hand X-ray images are divided into five different deep learning models are used to produce the feature maps.	-Not an end-to-end model since the bone age prediction is performed using Random Forest regressor technique.
Tang et al. [41]	CNNs	-Applies transfer learning to mitigate small training data.	-Does not perform any hyperparameter tuning.
Iglovikov et al. [1]	VGGNet	-Introduces image registration pipeline which includes hand segmentation, normalizing contrast, and key points detection.	-The model does not apply residual or feed-forward connections.
Chen et al. [42]	Inception-V3	-Applies attention guided method to localize three different regions using image-level labels.	-The system has applied gender information, which will give advantage for bone age prediction performance.
Reddy et al. [44]	CNNs	-Uses information of index finger only to train the CNNs architecture.	-Does not employ any pre-processing pipeline.
Marouf et al. [45]	CNNs	-Applies gender information in the model training process.	-Training accuracy and loss still fluctuate a lot for before the training has been ended.
Pan et al. [46]	InceptionResnet-V2	-Uses active learning to alleviate the annotation burden. -Applies transfer learning technique.	-The computational complexity is large due to ensembling process of hand masked segmentation and prediction module.
Hao and Li [47]	EfficientNet	-Applies pre-processing pipeline that include resizing, normalization, and data enhancement to remove bias and increase the number of training data.	-The experiment has included gender information which will give advantage for bone age prediction.

**Table 2:** AXNet network configuration.

Layer	Operator	Resolution	Channel	Kernel	Pool	Skip Connection
1	Convolution	288 × 288	32	3 × 3	No	No
2	Convolution	144 × 144	64	3 × 3	No	No
3	Separable convolution	144 × 144	128	3 × 3	No	No
4	Separable convolution	144 × 144	128	3 × 3	Yes	Yes
5	Separable convolution	72 × 72	256	3 × 3	No	No
6	Separable convolution	72 × 72	256	3 × 3	Yes	Yes
7	Residual	24 × 24	128	1 × 1	No	Yes
	Unit		256	3 × 3		
			512	1 × 1		
8	Attention	24 × 24	All	3 × 3	3 Downpool	Yes
	Unit		512		+	
					3 Upsample	
9	Residual	24 × 24	128	1 × 1	No	Yes
	Unit		256	3 × 3		
			512	1 × 1		
10	Attention	24 × 24	All	3 × 3	3 Downpool	Yes
	Unit		512		+	
					3 Upsample	
11	Residual	24 × 24	128	1 × 1	No	Yes
	Unit		256	3 × 3		
			512	1 × 1		
12	Attention	24 × 24	All	3 × 3	3 Downpool	Yes
	Unit		512		+	
					3 Upsample	
13	Residual	24 × 24	128	1 × 1	No	Yes
	Unit		256	3 × 3		
			512	1 × 1		
14	Attention	24 × 24	All	3 × 3	3 Downpool	Yes
	Unit		512		+	
					3 Upsample	
15	Separable convolution	24 × 24	728	3 × 3	No	No
16	Separable convolution	24 × 24	1024	3 × 3	Yes	Yes
17	Separable convolution	12 × 12	1536	3 × 3	No	No
18	Global pPool + Dense	1 × 1	2048	1 × 1	Yes	No

**Table 3:** Performance comparison of the hand masked segmentation models.

Method	$\bar{\mathrm{Acc}}$ (%)	IoU	Total No. of Parameters (Unit Register)	$H_{\mathrm{Dist}}$ (Pixels)
Stacked U-Net [48]	95.330	0.88967	3,036,802	40.848
PSPNet [49]	97.084	0.93094	27,896,000	7.625
DenseDeepLab V2 [50]	97.153	0.93234	110,054,344	3.558
FCN [51]	97.156	0.93253	134,393,428	2.747
DeepLab V1 [52]	97.198	0.93339	28,890,946	3.358
DeepLab V2 [53]	97.226	0.93405	71,419,720	3.606
DenseDeepLab V1 [50]	97.297	0.93565	40,900,546	3.368
SegNet [54]	97.709	0.94507	29,460,042	3.408
FC DenseNet [55]	97.741	0.94568	14,729,860	3.450
U-Net [56]	97.809	0.94739	31,032,834	2.897
DeepLab V3+ [57]	97.826	0.94778	41,253,888	3.791

**Table 4:** Performance comparison of the key-points detector models.

Method	MAE (Pixels)	MSE (Pixels${}^{2}$)	Total No. of. Parameters (Unit Register)
ResNet-50 [58]	0.12444	0.04091	23,577544
GoogleNet [59]	0.12410	0.13668	10,326,527
ShuffleNet V1 [60]	0.08183	0.02219	947,216
MobileNet V2 [63]	0.07564	0.02466	2,269,384
SqueezeNet [65]	0.06448	0.02091	739,600
MobileNet V3 [64]	0.05699	0.02098	3,795,832
ShuffleNet V2 [61]	0.05473	0.01999	5,395,104
LightCovidNet [8]	0.05028	0.01789	890,416
Xception-41 [66]	0.04913	0.01571	20,877,872
Xception-71 [66]	0.04776	0.01572	35,640,704
SPPCovidNet [7]	0.04389	0.01452	910,976
DenseNet-264 [67]	0.04138	0.01577	31,068,744
MobileNet V1 [62]	0.03563	0.01409	3,237,064

**Table 5:** Performance results of the AXNet and its benchmarked methods.

Method	MAE (Months)	MSE (Months${}^{2}$)	Total No. of Parameters (Unit Register)
ShuffleNet V1 [60]	15.728	372.575	936,457
Iglovikov et al. [1]	14.804	349.254	33,601,345
SqueezeNet [65]	14.164	311.783	735,939
VGG-19 [70]	14.028	307.416	38,911,041
MobileNet V3 small [64]	13.541	282.157	1,662,939
Hao and Li [47]	12.331	250.321	12,757,296
MobileNet V2 large [64]	12.307	242.316	3,786,865
ShuffleNet V2 [61]	12.010	226.951	5,380,761
MobileNet V2 [63]	11.394	213.454	2,260,417
Spampinato et al. [28]	11.173	205.067	95,116,161
Lee et al. [29]	10.972	220.759	5,973,224
MobileNet V1 [62]	10.886	190.349	3,229,889
DenseNet [67]	10.557	190.105	31,049,529
ResNet [58]	10.283	264.660	23,563,201
Inception V3 [71]	9.774	191.696	18,783,649
Pan et al. [46]	9.587	152.328	54,336,736
Xception-41 [66]	8.357	121.155	20,863,529
Zulkifley et al. [30]	8.200	121.902	20,863,529
AXNet	7.699	108.869	21,035,545

**Table 6:** Performance results of the ablation study.

Method	MAE (Months)	MSE (Months${}^{2}$)
AXNet without attention unit	8.357	121.155
AXNet without data normalization	8.219	119.240
AXNet	7.699	108.869

## Data Availability

The original dataset can be publicly downloaded from https://www.rsna.org/education/ai-resources-and-training/ai-image-challenge/rsna-pediatric-bone-age-challenge-2017. It was accessed on 3 November 2020.

## Abbreviations

The following abbreviations are used in this manuscript:

GP	Greulich–Pyle
TW	Tanner–Whitehouse
AXNet	Attention-Xception Network
ROIs	Regions of interest
ANN	Artificial Neural Networks
CNN	Convolutional Neural Network
GPU	Graphics Processing Unit
